# Evidence of SARS-CoV-2 in nasal brushings and olfactory mucosa biopsies of COVID-19 patients

**DOI:** 10.1371/journal.pone.0266740

**Published:** 2022-04-12

**Authors:** Carlotta Pipolo, Daniele Bottai, Emanuela Fuccillo, Eleonora Aronica, Fabio Bruschi, Antonio Mario Bulfamante, Luca Castellani, Maria Paola Canevini, Davide Chiumello, Sergio Ferrari, Carla Martinelli, Stefano Muttini, Alberto Priori, Alberto Maria Saibene, Chiara Spoldi, Delfina Tosi, Gianluigi Zanusso, Gaetano Pietro Bulfamante, Giovanni Felisati

**Affiliations:** 1 Department of Health Sciences, Università degli Studi di Milano, Milan, Italy; 2 Otorhinolaryngology Unit, Head and Neck Department, ASST Santi Paolo e Carlo Hospital, Milan, Italy; 3 Department of Clinical Sciences and Translational Medicine, University of Rome “Tor Vergata”, Rome, Italy; 4 Department of Neuropathology, Amsterdam UMC, University of Amsterdam, Amsterdam Neuroscience, Amsterdam, The Netherlands; 5 Epilepsy Centre, ASST Santi Paolo e Carlo Hospital, Milan, Italy; 6 Department of Anesthesia and Intensive Care, ASST Santi Paolo e Carlo Hospital, Milan, Italy; 7 Department of Neurosciences, Biomedicine and Movement Sciences, University of Verona, Verona, Italy; 8 Human Pathology and Medical Genetic, Cytogenetics and Molecular Pathology Unit, ASST Santi Paolo e Carlo Hospital, Milan, Italy; 9 Department of Anesthesia and Intensive Care, ASST Santi Paolo e Carlo Hospital, Milan, Italy; 10 “Aldo Ravelli”, Center for Experimental Neurotherapeutics, Milan, Italy; AUSL della Romagna, ITALY

## Abstract

The aim of the present study is to detect the presence of SARS-CoV-2 of patients affected by COVID-19 in olfactory mucosa (OM), sampled with nasal brushing (NB) and biopsy, and to assess whether a non-invasive procedure, such as NB, might be used as a large-scale procedure for demonstrating SARS-CoV-2 presence in olfactory neuroepithelium. Nasal brushings obtained from all the COVID-19 patients resulted positive to SARS-CoV-2 immunocytochemistry while controls were negative. Double immunofluorescence showed that SARS-CoV-2 positive cells included supporting cells as well as olfactory neurons and basal cells. OM biopsies showed an uneven distribution of SARS-CoV-2 positivity along the olfactory neuroepithelium, while OM from controls were negative. SARS-CoV-2 was distinctively found in sustentacular cells, olfactory neurons, and basal cells, supporting what was observed in NB. Ultrastructural analysis of OM biopsies showed SARS-CoV-2 viral particles in the cytoplasm of sustentacular cells. This study shows the presence of SARS-CoV-2 at the level of the olfactory neuroepithelium in patients affected by COVID-19. For the first time, we used NB as a rapid non-invasive tool for assessing a potential neuroinvasion by SARS-CoV-2 infection.

## Introduction

Smell dysfunction has been described as one of the clinical features in coronavirus disease 2019 (COVID-19) patients [1]. Even though rhinitis and rhinopharyngitis are not the more relevant symptoms of the disease [2], it is commonly understood that severe acute respiratory syndrome coronavirus 2 (SARS-CoV-2) usually enters through the nose and nasopharynx, giving rise to upper airway symptoms, and then to subsequently spread to the lower airways. The use of protective devices, although necessary, has resulted in similar rhinitis symptoms, headache or reduced work performance in health workers and may be mistaken for prodromal symptoms [3]. Smell dysfunction might be related to generic involvement of the nasal mucosa or specifically to involvement of the olfactory mucosa (OM) linked to SARS-CoV-2 neurotropism [1,4–7].

The presence of SARS-CoV-2 in the OM is a sign of involvement of the olfactory neuroepithelium. This is considered as an extracranial portion of the central nervous system (CNS), located in the upper portion of the nasal cavity [8]. SARS-CoV-2 can reach the olfactory bulb via the olfactory filaments and then spread to other brain areas [9–11]. This route uses anterograde axonal transport starting from the olfactory nerve (ON) [12,13], as has been described in experimental studies on transgenic mice [14].

Few autoptic studies have considered the presence of SARS-CoV-2 within the human CNS [15,16]. Bulfamante et al. [15] described the presence of particles attributable to SARS-CoV-2 virions in the medulla oblongata at the outer surface of its myelin sheath and in the neurons of the gyrus rectus. Furthermore, the same group found that patients severely affected with SARS-CoV-2 had severe damage to the ON, demonstrating a gradient of neural involvement from the ON to the gyrus rectus and finally to the brainstem, supporting the hypothesis that the viral invasion could start from the OM.

The presence and localization of SARS-CoV-2 have been demonstrated through quantitative RT-PCR (qRT-PCR) and immunohistochemistry (IHC) in selected brain regions of patients with COVID-19, revealing a high degree of astrogliosis and microgliosis in the olfactory bulb, and only minor infiltration by cytotoxic T lymphocytes [16]. Furthermore, it was recently observed by autoptic assessment that SARS-CoV-2 invasion involves primarily the ON, and can subsequently spread to other neuroanatomical areas, such as the respiratory and cardiovascular centers of the medulla oblongata [10].

Previous studies have shown that NB allows efficient samplings of the olfactory neuroepithelium, and it is currently used as a standard procedure for the diagnosis of Creutzfeldt-Jakob disease [17] and also for other neurodegenerative diseases such as Parkinson’s disease, multiple system atrophy, and idiopathic rapid eye movement sleep behavioral disorder, indicating that such a non-invasive procedure might be of high diagnostic value [18,19]. Furthermore, Nasal Brushing Sampling and Processing for Primary Ciliary Dyskinesia has been adapted for use during the COVID-19 Pandemic and risk management of COVID-19 patient interactions have been published [20,21].

The aim of our study was to demonstrate the presence of SARS-CoV-2 in the olfactory neuroepithelium of patients with COVID-19. To accomplish this goal, we explored the accuracy of two different sampling methods for detecting SARS-CoV-2 in the OM: a minimally invasive procedure termed nasal brushing (NB), and OM biopsy; the latter is currently considered the gold standard for evaluating the olfactory neuroepithelium [22,23].

## Materials and methods

### Ethical statement

The study was conducted between April 2020 and May 2020 at ASST Santi Paolo e Carlo Hospital in Milan, Italy, after receiving internal Institutional Review Board approval (Prot. n° 25/04/2020). The study followed the World Medical Association’s Declaration of Helsinki.

### Participants

The study was conducted in 17 participants with COVID-19 (diagnosis was made by two consecutive nasopharyngeal swabs positive for SARS-CoV-2 RNA) and admitted to the intensive care units of ASST Santi Paolo e Carlo Hospital and who were under deep sedation and mechanical ventilation because of severe pulmonary disease due to SARS-CoV-2 infection. Ten control participants were recruited from among voluntary medical staff and patients undergoing functional endoscopic sinus surgery (FESS) who had tested negative for SARS-CoV-2 and who had granted informed consent for donating their OM for the purpose of the present study. No sample size was calculated.

The participants histories were collected through electronic chart review. At the time of NB and OM biopsy, all 17 patients with COVID-19 were positive for SARS-CoV-2 molecular assay in nasal-oropharyngeal swab.

All 27 enrolled participants underwent NB; among them, only nine participants with COVID-19 and only four controls underwent OM biopsy. However, an insufficient number of cells was collected by NB from two participants; therefore, we decided to not include these patients in the study. The final cohort contained 15 patients with COVID-19 (13 men and 2 women; mean age, 59.7 years; median age, 58 years; age range, 34–73 years) and 10 healthy controls (4 men and 6 women; mean age, 40.5 years; median age, 40.5 years; age range, 24–60 years). All NBs and OM biopsies were performed by two different expert rhinologists (GF and CP).

### Patient and public involvement

The present work did not allow an active patient and public involvement in clinical research as part of its strategy, as the patients were under deep sedation, that was made necessary by their critical clinical situation.

### Olfactory mucosa sampling by nasal brushing and biopsy

NB was performed under endoscopic view using a 3-mm rigid nasal endoscope (Karl Storz SE & Co. KG, Tuttlingen, Germany) using a specifically designed flocked brush (FLOQBrush®; CopanItalia Spa, Brescia, Italy). The swab was inserted into the right nostril, reaching the upper portion of the middle turbinate and the upper septal mucosa, and gently rolled on the mucosal surface of the nasal vault [24]. At the end of the procedure, the brush was directly immersed in a 15-ml conical centrifuge tube containing the fixative Diacyte (Diapath S.p.a., Martinengo, Italy) for immunocytochemistry analysis [25]. OM biopsy specimens were collected from the contralateral side to which the NB had been performed to obtain preserved OM tissue. OM biopsies were collected by making a 2-mm incision from the upper-medial wall of the middle turbinate with an ophthalmic crescent blade and under endoscopic vision (0° nasal endoscope, Karl Storz). A single specimen of 3–4 mm^2^ was harvested using Hartmann straight ear forceps [26]. After the procedure, a 1-cm bioresorbable nasal dressing (Cellistyp, B. Braun Surgical S.A., Barcelona, Spain) was positioned in the superior meatus. The OM biopsies were placed in glutaraldehyde and in formalin to be processed for electron microscopy and histology, respectively.

The procedures were well tolerated, and only transient epistaxis was seen in a single participant who underwent OM biopsy, which was quickly resolved with standard placement of a local resorbable nasal dressing.

### Nasal brushing sample processing

The nasal swabs were processed as previously reported [25]. The samples were kept in fixative solution for 30 min at room temperature (RT) and treated for 15 min at RT with a mucolytic (*N*-acetylcysteine, 10 mg/ml final concentration) to remove nasal secretions and other debris.

Cells were then recovered by vortexing the swab for 1 min at RT. The swabs were discarded afterwards. The swab was removed and the tubes containing cell suspension were centrifuged at 123 ×*g* for 15 min at 4°C, and the resulting pellet was washed once in phosphate-buffered saline (PBS) solution. The pellets were resuspended in 100 μl PBS and counted with a hemocytometer. The appropriate number of cells (about 5,000–10,000) was spotted onto Superfrost Plus slides (Menzel-Gläser, Thermo Fisher Scientific), dried for 10 min at RT, and the slides were kept at 4°C or -20°C for IHC analysis.

### Nasal brushing immunocytochemistry

The slides were kept at RT for 5 min and hydrated in PBS for 10 min, and processed as previously described [27–29]. Briefly, the slides were incubated in blocking buffer (5% normal goat serum [NGS], 0.3% Triton X-100 in PBS) for 1 h at RT. We used the following primary antibodies against: TUJ1 (β-tubulin III, 3.3 μg/ml, MAB-10288, Immunological Science), NCAM (123C3) (sc-7326, 5 μg/ml, Santa Cruz Biotechnology Inc., or FLEX IR628, ready to use, Dako), and SARS-CoV-2 nucleocapsid antibody (3.3 μg/ml, GTX135361, GeneTex) diluted in incubation buffer (IB) containing 10% NGS and 0.3% Triton X-100 in 1× PBS, and the samples were incubated in a humid chamber overnight at 4°C. The next day, the slides were washed three times in PBS for 10 min each, and incubated with the appropriate secondary antibody: donkey anti-mouse Alexa Fluor 555 (IS20404, 2.5 μg/ml, Immunological Science) and donkey anti-rabbit Alexa Fluor 488 (IS20405, Immunological Science), diluted in IB, and kept in a humid chamber for 2 h at RT. The slides were washed in PBS three times for 10 min each, and nuclear counterstaining was performed using 300 μM 4′,6-diamidino-2-phenylindole (DAPI) in PBS for 10 min. Then, the slides were washed once in PBS for 10 min. Finally, the slides were mounted with Dabco (Molecular Probes, in 90% glycerol to 1 mg/ml), and a round coverslip was placed on the cell spot, allowed to dry, and sealed with nail polish. Images were obtained using a confocal microscope (FRET [fluorescence resonance energy transfer] Film, Nikon, Tokyo, Japan) and edited using GIMP-2.10 (The GIMP Development Team, 2019, https://www.gimp.org).

### Olfactory mucosal biopsy processing

OM biopsies were fixed in 10% paraformaldehyde for 72 h as recommended by SIAPEC-IAP guidelines (https://www.siapec.it/public/uploads/archiviodocumenti/Biosicurezza%20in%20anat%20pat%2025%20marzo%2020%20vers%2002_2020.pdf). Then, the samples were washed three times, dehydrated in alcohol solutions, and embedded in paraffin. Sections (3-μm thick) were mounted on Superfrost Plus® slides (Thermo Fisher Scientific) and processed for IHC.

### Olfactory mucosal biopsy immunohistochemistry

Immunohistochemical staining was performed using an automated immunostainer (Dako Omnis, Agilent Technologies®, Santa Clara, CA, USA); the slides were incubated for 1 h with the primary antibody following the manufacturers’ recommendations.

Neuronal cells were identified in DAB by monoclonal antibody (mAb) against β-tubulin III (PA5-85874, Invitrogen), while SARS-CoV-2 was detected by rabbit mAb against SARS-CoV-2 nucleocapsid (40143-R019, Sino Biological). The stains were visualized on a multi-station compound optical transmitted light microscope (Olympus®) and photomicroscope (Olympus®), and digitized using a NanoZoomer S60 slide scanner (Hamamatsu Photonics K.K., Hamamatsu, Japan). Images were obtained using NDP.view2 software.

### Electron microscopy

The COVID-19 patients’ olfactory biopsies were fixed in 2.5% glutaraldehyde in phosphate buffer and routinely processed for electron microscopy examination (JEOL JEM 1010 transmission electron microscope, JEOL, Tokyo, Japan).

## Results

### Detection of SARS-CoV-2 in nasal brushing samples of COVID-19 patients

**Fig 1:**
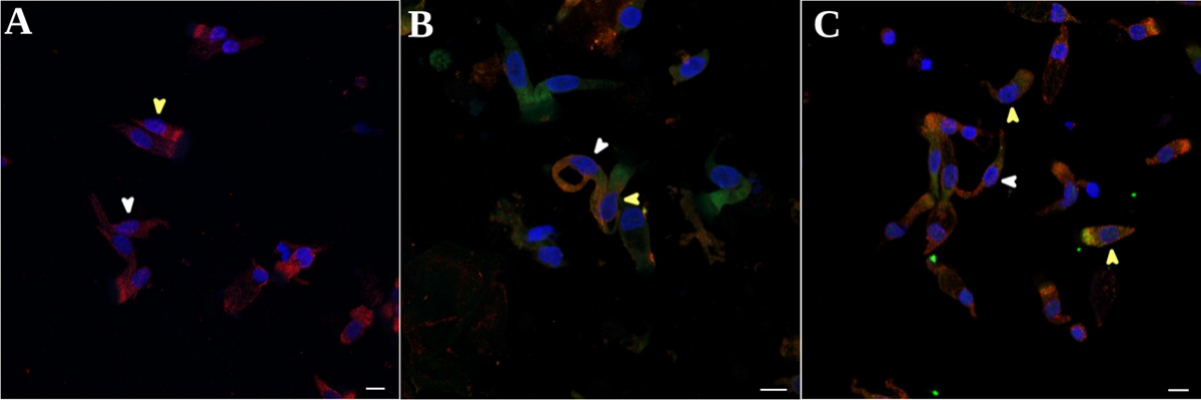
Immunofluorescence of a spotted sample of OM. (A) Healthy control (B) and (C) COVID-19 patients. In B and C, the green areas indicate immunoreactivity against the SARS-CoV-2 nucleocapsid. In B, the red areas represent immunoreactivity for NCAM; in A and C, the red areas indicate TUJ1 (β-tubulin III) immunoreactivity. In A, B, and C, the blue areas indicate DAPI staining of the nuclei. White arrowhead indicates neurons; yellow arrowheads indicate glia-like sustentacular cells or sensory MV cells. Scale bar: 10 μm in all panels.

Preliminary cell counts of NBs were 678,000 cells per single brushing from the 15 patients and 1,123,000 for the 10 controls. Two patients had to be excluded due to insufficient cells. The NBs from the controls were all negative for SARS-CoV-2 (Figs 1A and S1).

Immunocytochemical analysis showed immunoreactivity to the SARS-CoV-2 nucleocapsid in all examined NB samples (15 COVID-19 patients) (Fig 1B and 1C). SARS-CoV-2 positivity was found in non-neuronal cells and olfactory neurons; these cells were recognized and distinguished based on the distinct positivity to TUJ1 in the axonal hillock and based on their morphology (Fig 1B and 1C).

SARS-CoV-2 nucleocapsid immunoreactivity was also detected in other cell types, which had more rounded morphology and TUJ1 positivity in the apical portion of the cytoplasm, corresponding to supporting cells and microvillar (MV) cells (indicated by yellow arrowheads) or rounded cells positive for NCAM, likely basal cells (Fig 1B and 1C).

### Detection of SARS-CoV-2 in olfactory mucosa biopsies of COVID-19 patients

**Fig 2:**
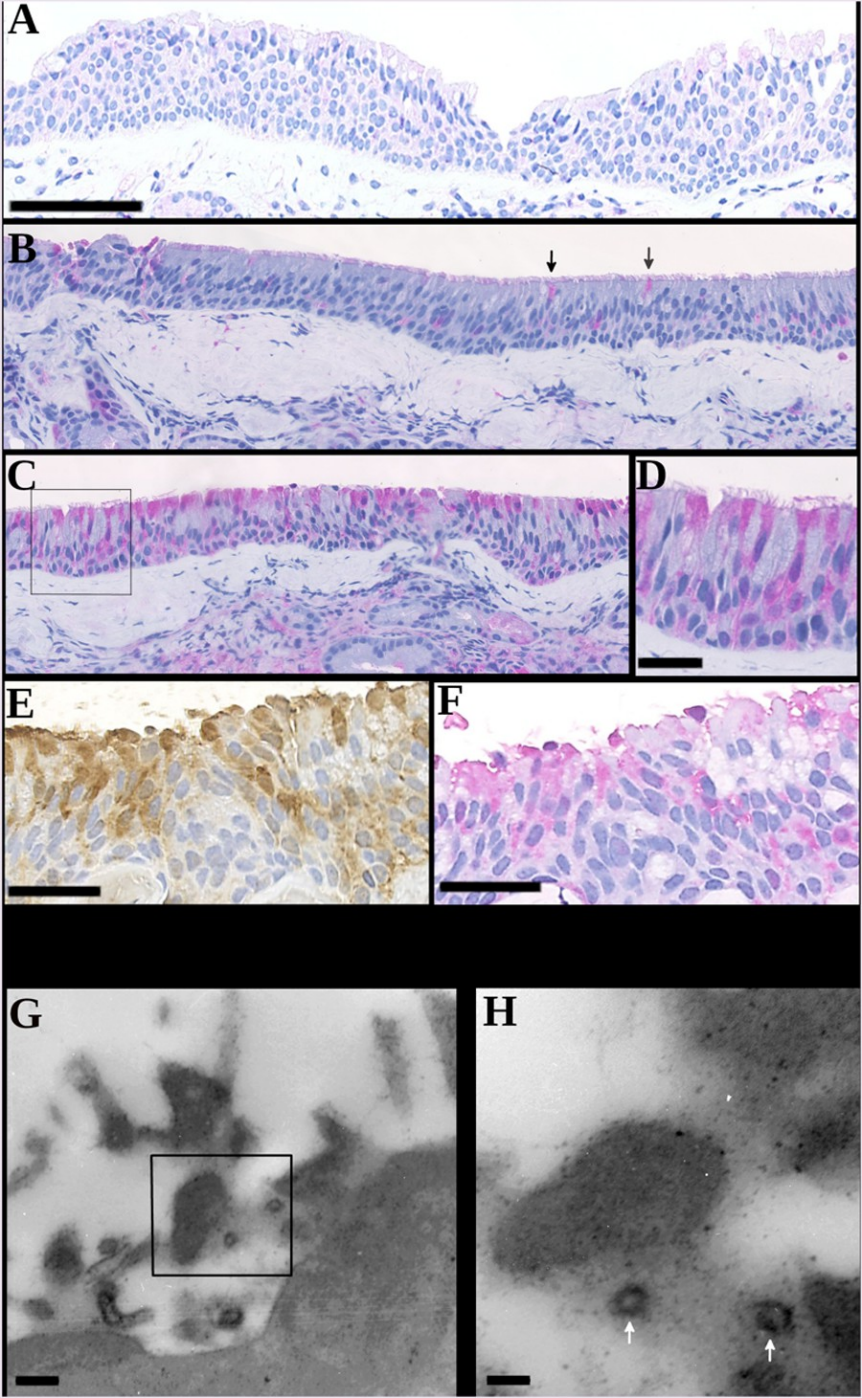
Olfactory biopsies in healthy controls and COVID-19 patients. (A) Healthy control. (B) Patient with low level of infection; arrow indicates the few neurons with an appreciable level of infection. C: Patient with high level of infection. D: Enlargement of a portion of C. E: Neuron immunostaining in the nasal mucosa. F: SARS-CoV-2 immunostaining in a consecutive section of E (A, B, C scale bar 100 μm, D, E, F scale bar 25 μm). G: Ultrastructural image of OM biopsy from a COVID-19 patient showing two extracellular SARS-CoV-2 particles in a ciliated cell (bar: 500 nm). H: In the inset, at higher magnification, the black particles show a recognizable SARS-CoV-2 structure (bar: 200 nm).

Nine participants underwent OM biopsy to confirm the results obtained by OM swabbing in the COVID-19 patients. Unlike NB, OM biopsy preserved the cell topography of the neuroepithelium, allowing more precise identification of each single SARS-CoV-2–positive cell [26]. SARS-CoV-2 immunoreactivities was detected in all nine examined OM biopsies from the COVID-19 patients, but not in the four OM biopsies of the controls (Fig 2A–2D).

SARS-CoV-2 positivity was unevenly distributed along the OM neuroepithelium, and the extent of OM involvement showed variability among patients. SARS-CoV-2 positivity was mainly observed in the cytoplasm of sustentacular cells, and in this case, to a lesser extent in the olfactory neurons, and the horizontal and globose basal cells (GBC) (Fig 2B–2D). Generally, SARS-CoV-2 positivity was comparable between the neuronal and non-neuronal cells. As expected, the sustentacular cells were distinct in the apical layers of the neuroepithelium, while the olfactory sensory neurons showed the typical bipolar, thin shape with the dendritic knob emerging in the nasal cavity (Fig 2D). Two consecutive slices immunostained for SARS-CoV-2 and β-tubulin III showed co-localization, emphasizing the involvement of the olfactory neurons (Fig 2E and 2F). Furthermore, we obtained ultrastructural evidence of SARS-CoV-2 in the OM biopsy. We observed a cluster of rounded, electron-dense viral particles surrounded by a membrane in the cytoplasm of the sustentacular cells (Fig 2G and 2H). Thus, immunohistochemical study of OM biopsy confirmed the pattern of SARS-CoV-2–positive cells observed in NB.

## Discussion

A large range of neurological manifestations has been widely described in patients with COVID-19 [1,4–7]. In particular, SARS-CoV-2 seems to enter the CNS through direct invasion of the OM, involving primarily the ON and subsequently spreading to other neuroanatomical areas, such as the respiratory and cardiovascular centers of the medulla oblongata [10]. Although SARS-CoV-2 involvement of the olfactory system has been shown at autopsy, this evidence is still lacking *in vivo* in patients infected by SARS-CoV-2. In the present study, we demonstrate the good efficiency not only of OM biopsy, but also of NB, in collecting olfactory neurons and supporting cells of the olfactory neuroepithelium, which were correctly identified in all NB samples with the exception of the two with insufficient cellularity.

Indeed, we showed a great accuracy in demonstrating SARS-CoV-2, as all examined NB swabs were positive, a result confirmed by the nine OM biopsy cases.

Considering that NB is a non-invasive, inexpensive, rapid, and painless procedure [24] and that our results reveal accuracy of this procedure for detecting SARS-CoV-2 invasion of the OM, it appears that NB can be used for diagnostic purposes related to the possible early detection of viral invasion of the olfactory neuroepithelium in COVID-19 patients.

There has never been a comparison between OM biopsy and NB as tools for studying the olfactory epithelium, and the present comparison has enabled the evaluation of the advantages and disadvantages of these two methods. In NB, the absence of the tissue cytoarchitecture is a limitation in the identification of cell types. Nevertheless, NB enables the examination of a relatively higher number of possibly positive cells, as shown when compared to OM biopsies. Biopsies are furthermore confined to a more limited harvest site, while NB reaches a wider region of the nasal vault. In our study, the higher presence of SARS-CoV-2–positive cells in the outer layers of the neuroepithelium confirms that harvesting of the deeper layers is unnecessary.

Moreover, there was comparable SARS-CoV-2 positivity in the non-neuronal cells and the olfactory neurons. These results are partially in contrast with other evidence, suggesting the possible entry of SARS-CoV-2 into the OM by exploiting receptors expressed in the non-neuronal cell types of the olfactory epithelium and that are absent from olfactory neurons [12,30], such as angiotensin-converting enzyme 2 (ACE2) and transmembrane protease serine 2 (TMPRSS2) [13]. SARS-CoV-2 invasion of the olfactory neurons might be subsequent to the primary involvement of non-neuronal cells, as we show that SARS-CoV-2 positivity is similar in olfactory neurons and in non-neuronal cells. The mechanisms of the passage between non-neuronal and neuronal cells remains unclear. Facilitation by neuropilin 1 (NRP1), which is abundantly expressed in the olfactory epithelium [31], could be possible, and seems to potentiate SARS-CoV-2 infectivity [32,33]. It must be emphasized that once olfactory neurons are positive for SARS-CoV-2, viral spreading to the olfactory bulb might occur via the olfactory pathway [14].

Furthermore, we may hypothesize that neuronal infection occurs during the early phases of their differentiation. Indeed, GBC express ACE2 receptors (albeit at lower levels than non-neuronal cell types of the olfactory epithelium) [30]. As the time of GBC-TA (transit-amplifying cells) differentiation to neurons is about 2–3 days in normal homeostatic conditions [34], we may postulate that the infection detected in a neuron could be the result of the previous infection of neuron precursors (note: Fig 2B and 2C where basal cells [most likely globose cells] are labeled with SARS-CoV-2 antibody). This might be compatible with SARS-CoV-2 viral tropism and cell damage induction [35].

The small study population represents one of the major limitations of this research. Moreover, we selected only patients with severe COVID-19; for this reason, the available anamnestic data are scarce and lack optimal correlation between the NB/OM biopsy results and the clinical symptoms, especially that regarding neurological data or the development of smell disorders.

## Conclusions

This is the first study to show the presence of SARS-CoV-2 viral nucleocapsid in the olfactory neurons and supporting cells of NB-harvested OM, from critically ill COVID-19 patients. These findings highlight the possible role of NB as a future diagnostic tool for detecting SARS-CoV-2 in neural tissue directly in contact with the environment and projecting to the olfactory brain areas. As NB is a simple and non-invasive procedure, it can represent an informative procedure for providing evidence of the early viral involvement of the CNS. A follow-up study is needed to evaluate the diagnostic value of OM brushing also in asymptomatic COVID-19 patients and in patients with mild symptoms.

## Supporting information

S1 Fig Immunofluorescence of a spotted sample of OM of healthy controls. Green areas indicate immunoreactivity against SARS-CoV-2 nucleocapsid; red areas indicate immunoreactivity against TUJ1, while blue areas represent DAPI staining of the nuclei. Scale bar: 10 μm in I is representative for all samples.
(TIFF)
