# Supplementary figures and images for: Evidence of SARS-CoV-2 in nasal brushings and olfactory mucosa biopsies of COVID-19 patients
Source: PLoS One. 2022 Apr 12;17(4):e0266740. doi: 10.1371/journal.pone.0266740 (PMC9004784; doi:10.1371/journal.pone.0266740)

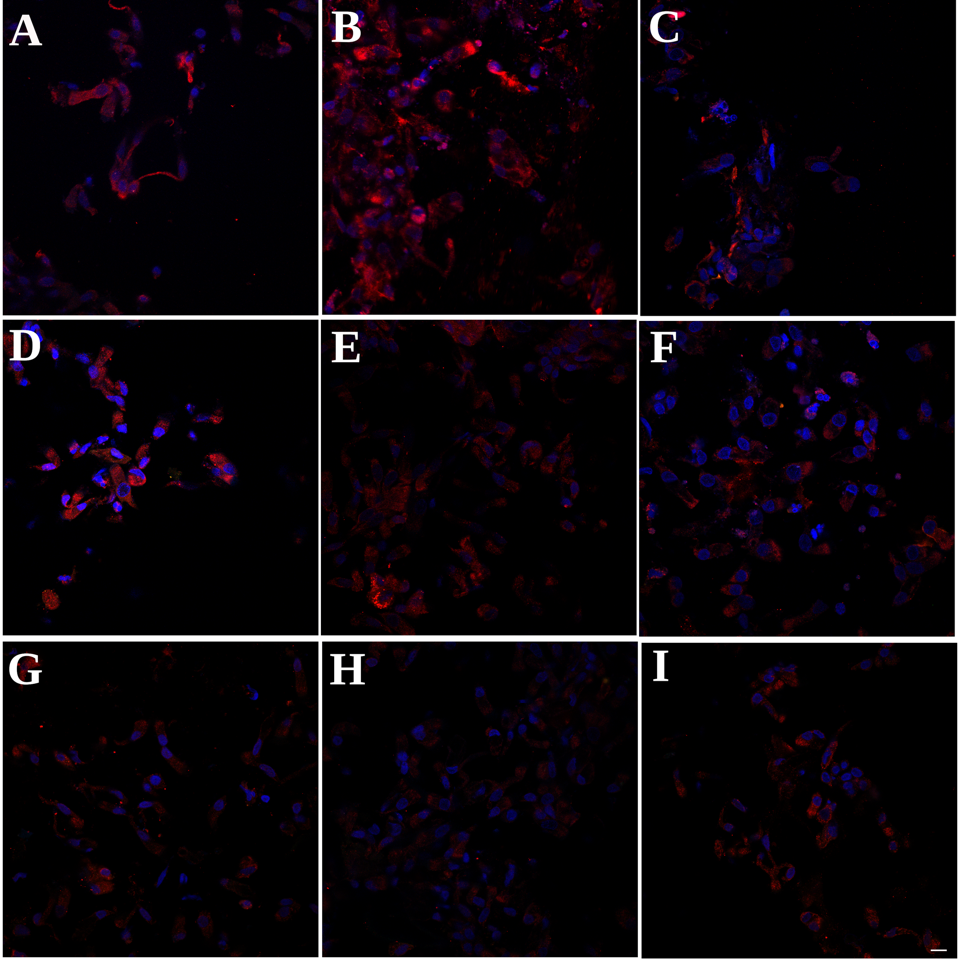

Supplement: S1 Fig — Green areas indicate immunoreactivity against SARS-CoV-2 nucleocapsid; red areas indicate immunoreactivity against TUJ1, while blue areas represent DAPI staining of the nuclei. Scale bar: 10 μm in I is representative for all samples. (TIFF) [file pone.0266740.s001.tiff]
